# Variable host responses mediate host preference in marine flatworm−snail symbioses

**DOI:** 10.1371/journal.pone.0247551

**Published:** 2021-03-02

**Authors:** Juhyung Lee, Timothy M. Davidson, Mark E. Torchin

**Affiliations:** Smithsonian Tropical Research Institute, Balboa, Ancon, Republic of Panama; Evergreen State College, UNITED STATES

## Abstract

Host preference of symbionts evolves from fitness trade-offs. However, it is often unclear how interspecific variations in host response traits influence this evolutionary process. Using the association between the polyclad flatworm *Paraprostatum echinolittorinae* and its intertidal snail hosts on the Pacific Coast of Panama, we assessed how a symbiont’s host preference is associated with varying host defenses and post-infestation performances. We first characterized the prevalence and intensity of worm infestation in five snail hosts (*Tegula pellisserpentis*, *Nerita scabricosta*, *N*. *funiculata*, *Planaxis planicostatus*, *and Cerithium stercusmuscarum*). We then used manipulative experiments to test flatworm’s host choice, hosts’ behavioral rejection of flatworms, and hosts’ growth and survival following the infestation. In the field, flatworms were orders of magnitude more prevalent and dense in *T*. *pellisserpentis*, *N*. *scabricosta*, *N*. *funiculata* than *P*. *planicostatus* and *C*. *stercusmuscarum*, although the three former hosts were not necessarily more abundant. The results from our laboratory host selection trials mirrored these patterns; flatworms were 3 to 14 times more likely to choose *T*. *pellisserpentis*, *N*. *scabricosta*, *N*. *funiculata* over *P*. *planicostatus* and *C*. *stercusmuscarum*. The less preferred hosts frequently rejected flatworms via mantle contractions and foot withdrawals, which reduced the infestation rate by 39%−67%. These behaviors were less frequent or absent in the preferred hosts. Flatworm infestation variably influenced host performances in the field, negligibly affecting the growth and survival of *T*. *pellisserpentis* and *N*. *funiculata* but reducing the growth of *P*. *planicostatus*. Flatworms thus preferred less defended hosts that can also support higher worm densities without being harmed. Stable isotope analysis further revealed that flatworms are unlikely to feed on snail tissues and may live as a commensal in their preferred hosts. Our study demonstrates that host response traits can modulate a symbiont’s host choice and calls for more explicit considerations of host response variability in host preference research.

## Introduction

In symbiotic interactions [1], the identity of partner species can alter the costs and benefits for hosts and symbionts, leading to differences in the degree to which the interactions are beneficial, neutral, or antagonistic [2]. Symbionts perform differently and achieve variable fitness levels in different hosts [3–5]. Also, the performance, survival, and reproductive success of hosts depend strongly on the species and phenotypes of associated symbionts [6, 7].

Conceptual theories posit that this partner-dependence is central to the evolution of host-preference in symbioses [8]. Host preference may evolve from host-dependent fitness tradeoffs that are determined by the relative availability and quality of different host species [8–11]. For instance, symbionts could gain maximum reproductive success and fitness by associating with more abundant hosts that they can frequently encounter and search efficiently [10–12]. Alternatively, symbionts could choose higher quality hosts in which their adults and offspring can achieve maximum performance or fitness level [8, 9]. The latter prediction is increasingly supported by empirical findings that symbionts perform better and achieve higher fitness levels in their preferred hosts [5, 13, 14].

Several different factors could modulate the success of symbionts in different host species and contribute to their host preference. These include but are not limited to life-history traits of symbionts and hosts (e.g., life-stage, reproductive status, and body size) [15, 16], environmental conditions [17], ecological interactions with natural enemies or competing species [16, 18], and host response traits (e.g., behavioral or immunological defense against symbionts) [11, 19, 20]. Assessing the relative importance of these drivers and their complex interactions is challenging but vital to understand the evolution and maintenance of host preference.

In this study, we evaluate how host response traits modulate host preference during symbiotic interactions. Host responses to symbionts, both adaptive and nonadaptive, could affect symbionts’ success in different hosts [21]. For example, hosts may variably regulate a symbionts’ performance and abundance with their behavioral or immunological defense, as demonstrated in several symbiotic relationships [22–24]. Different host performance and survival upon parasite or pathogen infection may also alter the symbionts’ transmission and fitness [25]. Such findings lead us to predict that interspecific variations in host response traits would significantly influence a symbionts’ host choice. However, empirical evaluations of the prediction thus far have been largely restricted to a few study systems (e.g., avian brood parasitism and parasitoid-insect host interaction) [19, 26, 27].

We examined the host preference of symbiotic marine flatworms (Platyhelminthes: Turbellaria: Polycladida) and their varying effects on host organisms. Polyclads are predominantly free-living [28]. However, several species, mostly belonging to the suborder Acotylea, live as ecto-symbionts of marine invertebrates, including corals, molluscs, crustaceans, and echinoderms [28–31]. Symbiotic polyclads are potentially useful models for testing ecological and evolutionary mechanisms underlying host preference in marine symbionts, owing to their ability to utilize multiple host species and greater ease of manipulation under a laboratory setting [30, 32].

**Fig 1:**
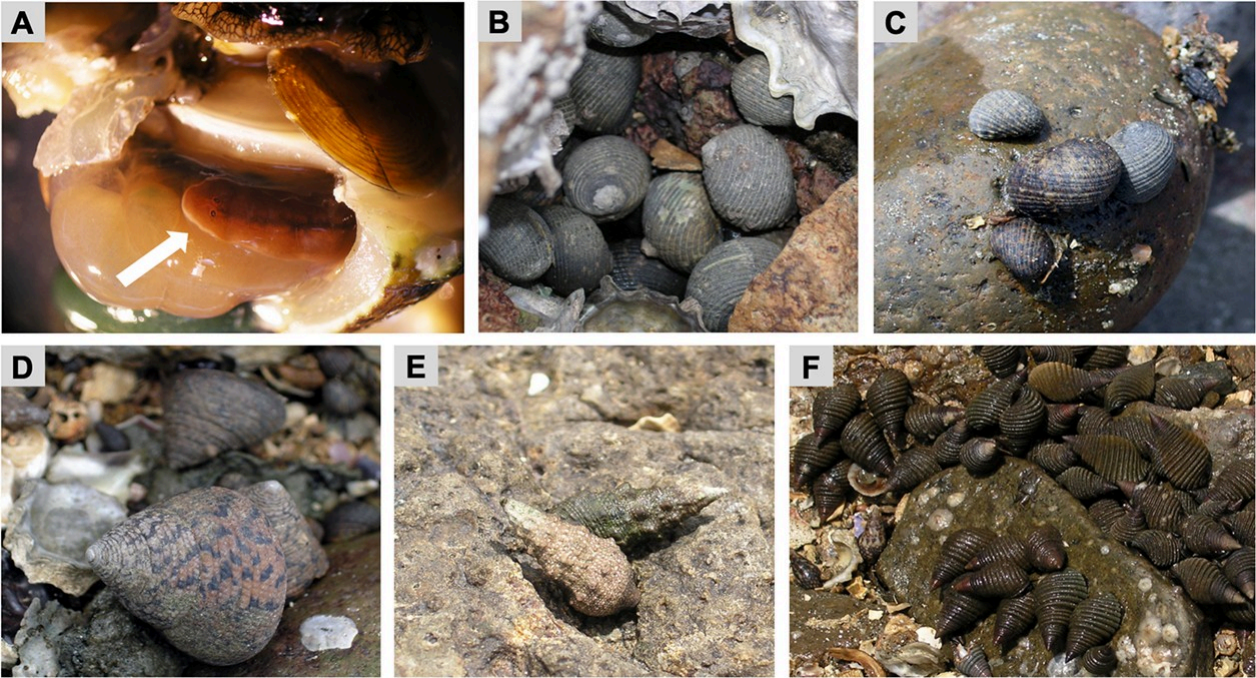
Symbiotic flatworm *Paraprostatum echinolittorinae* (Platyhelminthes: Turbellaria: Polycladida) and its snail hosts. (A) A flatworm inside the mantle cavity of snail host (*Tegula pelllisserpentis*), as indicated by a white arrow. Five species of intertidal snails common on the Pacific Coast of Panama including (B) *Nerita scabricosta*, (C) *N*. *funiculata*, (D) *Tegula pelllisserpentis*, (E) *Cerithium stercusmuscarum*, and (F) *Planaxis planicostatus*.

The flatworm *Paraprostatum echinolittorinae* is one of several Acotylean polyclads known to infest marine gastropods (e.g., snails and limpets) [28, 30, 33] and is found along the entire Pacific Coast of Central America [33]. In coastal Panama, the species is associated with snail species belonging to four different families (i.e., Tegulidae, Neritidae, Planaxidae, and Cerithiidae). The flatworm usually inhabits the host mantle or branchial cavity (Fig 1A). While its ecological effects on snails have not been determined, our initial laboratory and field observations suggested that snail hosts may respond variably to flatworm infestation. The pilot infestation trials revealed that not all snails readily accept infesting flatworms, and some species (e.g., *P*. *planicostatus*) will actively reject the worms via mantle contractions and foot withdrawals. We further observed that, whereas most snails rarely host more than a single or a few worms, some species will frequently support much higher worm loads (e.g., ~ 39 worms per snail in *T*. *pellisserpentis*).

We hypothesized that snail hosts would differentially respond to flatworm infestation and that this host response variability would mediate the worm’s host choice. Specifically, we predicted a) snail hosts would reject and repel infesting flatworms to a varying degree, b) snails showing a stronger rejection behavior would perform more poorly upon flatworm infestation, and c) flatworms would prefer hosts that are less likely to reject and be harmed by the infestation. To test our hypothesis, we first conducted field surveys to quantify flatworm prevalence and abundance in five common snail hosts (*Tegula pellisserpentis*, *Nerita scabricosta*, *Nerita funiculata*, *Planaxis planicostatus*, and *Cerithium stercusmuscarum*; Fig 1B–1F). We then performed two separate experiments under a laboratory condition, each investigating flatworms’ host choice and snails’ behavioral rejection of infesting worms. Finally, we used a field experiment to assess whether flatworms variably influence the growth and survival of their preferred and less-preferred snail hosts.

## Materials and methods

### Flatworm prevalence and infestation density in snail hosts

To examine the prevalence and density of flatworm infestation in different snail species, we conducted field surveys during January 2012. Snails were collected in the rocky intertidal near the Smithsonian Tropical Research Institute’s Punta Culebra Nature Center (PCNC; 8°54’43.54"N, 79°31’46.17"W) on Naos Island at the Pacific entrance of the Panama Canal. We collected *T*. *pellisserpentis* (n = 87), *N*. *funiculata* (n = 97), and *P*. *planicostatus* (n = 99) from mid-intertidal rocks, and *N*. *scabricosta* (n = 101) from high-intertidal rocks. In addition to these species, we collected 114 individuals of *C*. *stercusmuscarum* from high-intertidal tide pools.

Snails were brought back to the laboratory, where we measured shell length, width, and height to the nearest 0.01 mm and recorded blotted wet mass (BWM, to 0.0001 g). Live flatworms were removed by carefully crushing snail shells and dissecting them from the snail mantle. We counted the number and measured BWM of all flatworms that were found within each dissected snail. We also approximated the volume of mantle cavity in four snail species (*T*. *pellisserpentis*, *N*. *scabricosta*, *N*. *funiculata*, and *P*. *planicostatus*) by measuring the change in snail wet mass before and after removing all the water from the mantle (by gently pushing the snail operculum into the shell). For each species, this procedure was repeated with six different-sized individuals, covering a natural range of body size. Change in water mass (g) was converted into water volume (cm^3^; 0.98 cm^3^ per 1 g seawater) for the approximation of snail mantle volume.

To quantify the relative abundance of snail species, we conducted additional field surveys during June 2012 at PCNC. Four transects (40−45 m in length, > 3 m apart) were placed perpendicularly to the shoreline, from the high intertidal to the low intertidal zone. Along each of four transects, we placed two quadrats (1 m^2^) per each of three intertidal zones (high, mid, and low; total n = 8 quadrats per zone) and counted all snails found within each quadrat. *C*. *stercusmuscarum* was generally limited to tide pool areas within the high intertidal zone, so we laid four additional transects (10−12 m in length, > 2 m apart) with two quadrats each across tide pool areas.

### Host selection behaviors in flatworms

We performed a laboratory experiment to test the host selection behaviors of symbiotic flatworms. Snails were collected from PCNC between February and May 2012. For larger snail species (*T*. *pellisserpentis*, *N*. *scabricosta*), we collected smaller individuals to control for the potential confounding effect of host body size (Shell length; *T*. *pellisserpentis* = 16.7−19.6 mm, *N*. *scabricosta* = 12.6−15.2 mm, *N*. *funiculata* = 11.7−14.3 mm, *P*. *planicostatus* = 19.3−23.8 mm, *C*. *stercusmuscarum* = 18.6−24.1 mm). In the laboratory, we sacrificed multiple individuals of each of five snail species to extract live flatworms. Due to the scarcity of flatworm infestation in *P*. *planicostatus* and *C*. *stercusmuscarum*, experimental flatworms were all obtained from *T*. *pellisserpentis*, *N*. *scabricosta*, and *N*. *funiculata*. Flatworms that were collected from different host species were placed in separate containers until further use. To ensure that all snails used during the experiment were free of flatworms, we flushed the mantle of all study snails with Flatworm Exit (Salifert^®^, diluted to 0.2% with seawater) using a needleless syringe. This solution is used in commercial aquariums and in experimental studies for removing polyclad flatworms and is purportedly non-toxic to other marine invertebrates and fishes [34]. After 30 minutes of the exposure, the snails were transferred to an aerated seawater tank and held for at least one week prior to experimental trials.

Host-selection trials were conducted in a cylindrical glass bowl (diameter = 25 cm), containing one individual of each of five snail species (*T*. *pellisserpentis*, *N*. *funiculata*, *N*. *scabricosta*, *P*. *planicostatus*, and *C*. *stercusmuscarum*). Prior to each trial, we fixed experimental snails upside-down to the bottom of the bowl using cyanoacrylate adhesive (Krazy Glue ®) in order to immobilize them and to better observe whether or not flatworms have entered the snail cavity. Snails were positioned so that they were equidistant from each other and from the center of the bowl. Once the glue had dried, we gently flushed the experimental bowl several times with seawater and waited at least 30 minutes before introducing flatworms. At the onset of each trial, we introduced 9 flatworms (3 worms collected per snail species; *T*. *pellisserpentis*, *N*. *scabricosta*, and *N*. *funiculata*) to the center of the bowl and monitored their host choice for 45 minutes. We considered the host was “chosen” when the flatworm, for the first time, entered the host aperture opening or mantle (irrespective of worms being subsequently repelled by snail hosts or voluntarily exiting the hosts afterward). We calculated the infestation probability for each snail species as a proportion of introduced worms that selected the experimental snail within a 45-minute timeframe. This trial was repeated 18 times throughout the study, with different individuals of snails and flatworms.

### Host rejection of flatworm infestation

Our preliminary observations suggested that snails do not actively avoid flatworms (e.g., fleeing from approaching worms). However, some snails retracted their foot and contracted their mantle when flatworms attempted to enter their mantle cavity. This often prevented flatworms from entering snails’ mantle cavity, suggesting the behavior may be defensive. We experimentally compared the frequency of this rejection behavior among five snail host species. In the laboratory, we placed an individual snail upside-down (with the aperture upwards) on a glass petri dish (diameter = 7 cm) and then placed a flatworm directly into the shell aperture. This approach allowed us to observe host behaviors with greater ease when flatworms entered their cavity. Flatworm infestation caused a similar behavioral response in snail hosts whether they be placed in an upside-down or upright position. We recorded the presence or absence of host rejection behavior over a 3-minute period. We then determined whether experimental worms have successfully entered the hosts by visually inspecting the shell surface and aperture of snail subjects. Host and flatworm responses were classified into four categories as follows: a) the host rejects and repels the worm, b) the host rejects the worm, but the worm successfully infests the host, c) the host does not reject the worm, and the worm does not infest the host either, and d) the host does not reject the worm, and the worm successfully infests the host. For each snail species, we repeated this trial 18 times using different individuals of snails and flatworms.

### Host growth and survival following worm infestations

To examine the effect of flatworm infestation on host growth and survival, we conducted a field caging experiment with the subset of host species (*T*. *pellisserpentis*, *N*. *funiculata*, and *P*. *planicostatus*). We chose these snails because they overlap in their natural habitat (i.e., mid-intertidal zone) and represent host species that flatworms show high (*T*. *pellisserpentis*), moderate (*N*. *funiculata*), and low (*P*. *planicostatus*) preference. Snails were collected at PCNC in April 2012. We measured snails’ shell dimensions and BWM as above and randomly assigned them into two groups (n = 18 per treatment group per species): without flatworms and with flatworms. We also sacrificed multiple individuals of *T*. *pellisserpentis* and *N*. *funiculata* to obtain flatworms. To ensure that all snails were free of flatworms before being assigned to different treatment groups, we flushed the mantle of experimental snails with Flatworm Exit solution as above. The snails that had been assigned to the flatworm treatment group were re-infested with flatworms by placing haphazardly chosen worms (i.e., irrespective of original host species) directly on the aperture of each snail. For each snail species, we manipulated flatworm density differentially to simulate the natural infestation density (no. worm per snail, *T*. *pellisserpentis* = 8, *N*. *funiculata* = 4, and *P*. *planicostatus* = 2). The worm densities used here represented a high density that is less common in the field. Still, because we selectively used smaller flatworms (0.4−0.8 mg), the combined biomass of flatworms infesting each snail was well within the range of natural infestation. To track snail shell growths, we marked the margin of each snail shell aperture with paint covered with a thin layer of superglue. At the beginning of the experiment, the size of snails did not vary between the two groups for all three species (SL ± SE [mm]; *T*. *pellisserpentis*, without flatworm = 13.99 ± 0.76 mm, with flatworm [8 worms] = 14.02 ± 0.75 mm, *t*_34_ = − 0.026, *P* = 0.980; *N*. *funiculata*, without flatworm = 11.43 ± 0.13 mm, with flatworm [4 worms] = 11.43 ± 0.14 mm, *t*_34_ = − 0.038, *P* = 0.970; *P*. *planicostatus*, without flatworm = 15.70 ± 0.30 mm, with flatworm [2 worms] = 15.69 ± 0.30 mm, *t*_34_ = 0.037, *P* = 0.971).

In the mid-intertidal zone at PCNC, we cleared a 3.5 m × 1.0 m area of rocks to create an even surface and installed 36 cages, arranging them into a 3 × 12 block formation. We constructed each cage (S1 Fig) using a single hexagon-shaped brick (width = 19 cm, height = 13 cm) with a threaded hole (hole diameter = 14.5 cm). We filled the hole with similarly sized rocks to create interstitial spaces for snails and then covered the top and bottom openings with a wire mesh (0.75 cm × 0.75 cm). We randomly assigned individual snails of each species irrespective of the treatment type (i.e., with or without flatworm) to each of the 36 cages. Therefore, a single cage held three snail individuals, each of different species. This “cage sharing” was necessitated to minimize the physical disturbance in our study site. The three species co-occur naturally, and the density and size ranges of the caged snails were commonly observed in the field.

We maintained the experiment by cleaning all cages every other day and recorded snail mortality during each visit. After 52 days, we terminated the experiment and measured shell growth (i.e., the maximum distance between the newly formed margin of shell aperture and the outer margin of the painted surface) of all surviving snails. Tissues were extracted from the shells, and the sex of each individual was determined. Snail tissues were oven-dried at 65°C for 36 hours to obtain dried tissue mass (DTM) as a more robust measure of growth. We calculated the growth rate as the proportional increase in DTM. The initial DTM of each experimental snail was estimated using empirically determined relationships between external shell dimensions and DTM as in Byers [35]. We derived this relationship by measuring the shell length and weighing the DTM of snails over a range of sizes (n = 30) and regressing the DTM against the shell length (for all species, *r*^2^ > 0.90).

### Stable isotope analysis of host and flatworm tissues

To determine whether flatworms directly feed on host tissue, we analyzed the stable isotopic signatures of snail and flatworm tissues. We sampled tissues from freshly collected *T*. *pellisserpentis*, *N*. *scabricosta*, and *N*. *funiculata* and flatworms infesting each snail species (apart from the studies described above). Due to the difficulty of obtaining a sufficient amount of flatworm tissues from *P*. *planicostatus* and *C*. *stercusmuscarum*, we excluded the two snail species from the study. We sampled the gill and muscle tissues (i.e., mantle and foot) for snails and the whole body for flatworms. All samples were washed with distilled water, dried at 60°C for 36 hours, and ground to a fine powder prior to analysis. For each tissue category, we analyzed four to eight separate samples (ca. 2 mg) using an elemental analyzer (Flash HT) and Delta V Advantage isotope ratio mass spectrometer (CF-IRMS; Thermo Scientific, Bremen, Germany) in the Stable Isotope Laboratory of the Smithsonian Tropical Research Institute, Panama. Stable isotope values are expressed in delta notation as parts per thousands (‰) deviations from the standard using the following equation:

$$\delta Χ=(R_{sample}/R_{standard}-1)\times 1000$$

where *X* is ^13^C or ^15^N, and *R* is the ratio between ^13^C and ^12^C or ^15^N and ^14^N. For standard reference materials, we used Pee Dee Belemnite for carbon and atmospheric nitrogen N_2_ for nitrogen. Each trophic transfer from prey to consumers is typically represented by the average increase of δ ^15^N values by ca. 3.4‰ and δ ^13^C values by ca. 1‰ [36–38]. In general, δ ^15^N is used to determine the difference in trophic level, whereas δ ^13^C is used to distinguish between primary carbon sources (e.g., phytoplankton and macroalgae) [36].

### Statistical analysis

We used a generalized linear model (GLM) with a logit link function (i.e., logistic regression), to compare a) the prevalence of flatworm infestation (infested vs. non-infested) in different snail hosts, b) the frequencies of different host response types during the host rejection experiment, and c) the snail mortality (live vs. dead) during the field experiment. We used a GLM with a quasi-Poisson error distribution for over-dispersed data [39] to compare the density of flatworm infestation in different snail hosts. We used a linear mixed model (LMM, R package lme4) to compare the probability of worm infestation for different snail species (square root-transformed to achieve normality and homogeneity of variance) during the host selection experiment. We treated snail species as a fixed factor and trial ID as a random factor. The significance of snail species as a fixed factor was assessed using Type III ANOVA with Satterthwaite approximations to determine denominator degrees of freedom (R package lmerTest). Finally, we used a generalized linear model with a Gaussian link function to analyze snail growth (i.e., shell growth and % increase DTM) during the field experiment. For multiple comparisons among GLMs and LMM outputs, we used Holms-corrected *P* values (R package multcomp). We conducted all analyses using R (version 3.5.1; R Core Team 2018).

### Ethics statement

A field collection permit (permit no. DGOMI-PICFC-N°23) was issued by Panama’s Autoridad Nacional del Ambiente, and Autoridad de Recursos Acuáticos de Panamá. We also followed all applicable guidelines for the care and use of animals established by the Smithsonian Institution during our laboratory and field work.

## Results

### Flatworm prevalence and infestation density in snail hosts

**Fig 2:**
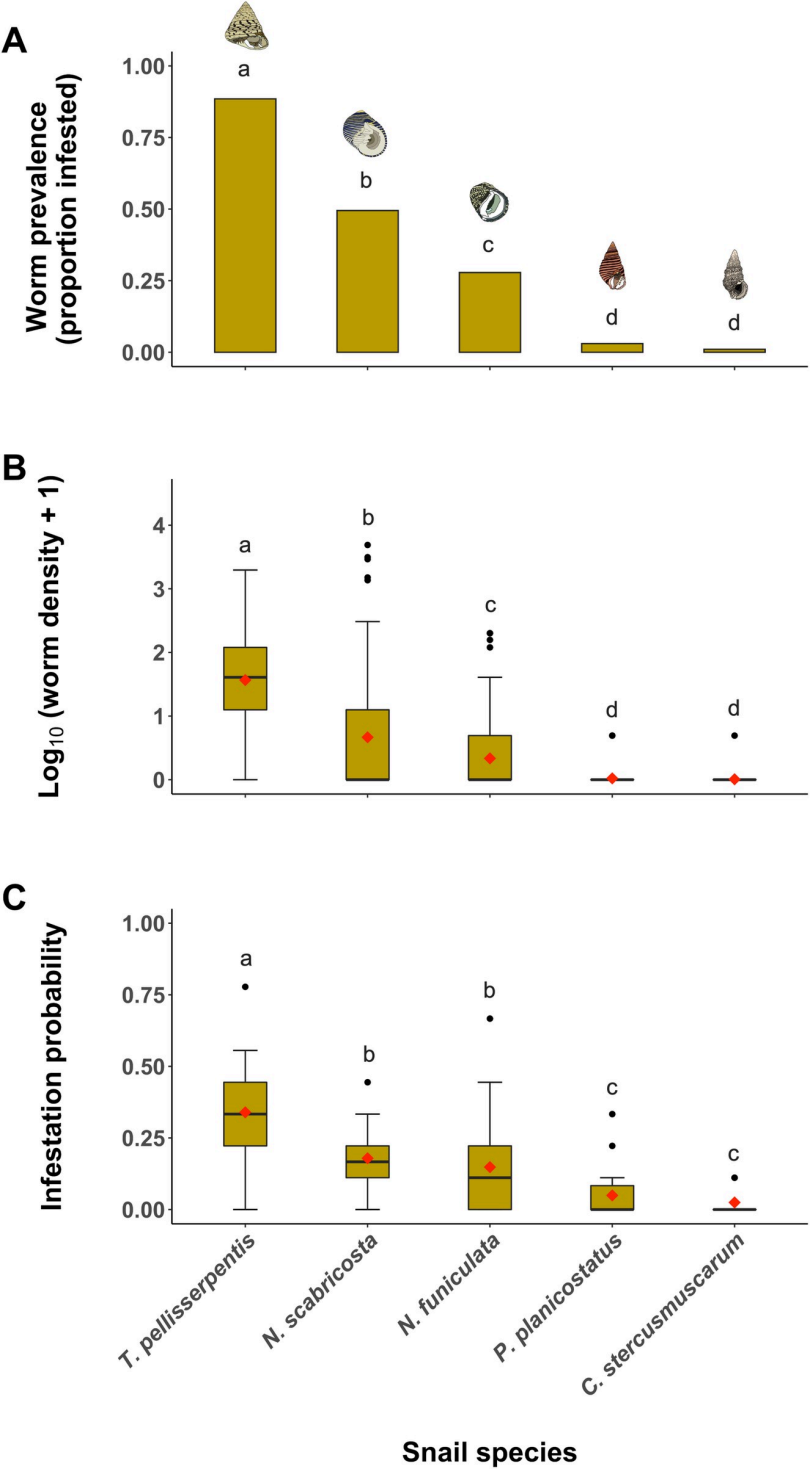
Prevalence and density of flatworm infestation in the field and flatworms’ host choice in the laboratory. (A) Proportion of snail individuals infested by flatworms in the field, (B) box plot of flatworm infestation density (no. worm per snail) in the field, and (C) box plot of infestation probability (i.e., a proportion of individual worms selecting a particular host species) measured during the host selection experiment. In box plots, mean values for different snail groups are represented by red diamond symbols. Different letters indicate a significant difference based on post hoc multiple comparisons (Holms-corrected P < 0.05).

We dissected 498 snails and recovered a total of 844 individual flatworms. The prevalence of flatworm infestation in each host species was: *T*. *pellisserpentis* = 89%, *N*. *scabricosta* = 50%, *N*. *funiculata* = 28%, *P*. *planicostatus* = 3%, *C*. *stercusmuscarum* = 0.01%; and the mean density of worm infestation (no. worms per snail ± 1 SE) was: *T*. *pellisserpentis* = 5.7 ± 0.6, *N*. *scabricosta* = 2.6 ± 0.7, *N*. *funiculata* = 0.8 ± 0.2, *P*. *planicostatus* = 0.03 ± 0.02, *C*. *stercusmuscarum* = 0.01 ± 0.01. Both the prevalence (Logistic regression; χ^2^ = 354.8, df = 4, *P* < 0.0001; Fig 2A) and mean density (quasi-Poisson GLM; F_[__4__,475]_ = 51.1, *P* < 0.0001; Fig 2B) of flatworm infestation varied significantly among snail hosts. Morphometric measurements for each snail species including shell length, BWM, and approximated cavity volumes are summarized in Table 1.

**Table 1:** Morphometrics, habitat, and field abundance of snail hosts.

		*T*. *pellisserpentis*	*N*. *scabricosta*	*N*. *funiculata*	*P*. *planicostatus*	*C*. *stercusmuscarum*
Shell length (mm)		30.35 ± 0.88 mm	25.87 ± 0.38 mm	11.26 ± 0.25 mm	18.10 ± 0.38 mm	20.32 ± 0.40 mm
BWM (g)		14.27 ± 0.82 g	8.89 ± 0.32 g	0.72 ± 0.04 g	1.15 ± 0.06 g	1.01 ± 0.05 g
Approximated mantle volume (cm^3^)		1.03 ± 0.25 cm^3^	0.33 ± 0.02 cm^3^	0.14 ± 0.02 cm^3^	0.13 ± 0.02 cm^3^	-
Snail density (no. individual / m^2^)	Tide pool (n = 8)	0 ± 0 (0%)	0 ± 0 (0%)	46.6 ± 11.7 (100%)	0 ± 0 (0%)	75.5 ± 16.7 (100%)
	High zone (n = 8)	0 ± 0 (0%)	11.6 ± 4.5 (75%)	8.8 ± 4.6 (50%)	23.6 ± 10.2 (50%)	6.9 ± 6.9 (13%)
	Mid zone (n = 8)	1.1 ± 0.5 (63%)	2.6 ± 1.7 (25%)	6.8 ± 3.0 (50%)	27.1 ± 10.1 (63%)	1.5 ± 1.5 (13%)
	Low zone (n = 8)	1.8 ± 0.7 (50%)	2.3 ± 2.3 (13%)	32.1 ± 11.8 (63%)	25.8 ± 9.6 (75%)	0 ± 0 (0%)
	**Total (n = 32)**	**0.7 ± 0.2 (28%)**	**4.1 ± 1.5 (28%)**	**23.6 ± 5.1 (66%)**	**19.1 ± 4.6 (47%)**	**21.0± 7.1 (31%)**

Results summarizing morphometric measurements (i.e., shell length, blotted wet mass, and approximated mantle volume) and field density (no. individual per m^2^) of five snail hosts. All surveys were conducted between January and June 2012 at the Smithsonian Tropical Research Institute’s Punta Culebra Nature Center (PCNC), Panama. For each species, shell length and BWM were measured from 87 to 101 individual snails, and mantle volumes were approximated using 6 different-sized individuals, covering a natural range of body size. Snail density was measured across four distinct zones within the intertidal habitat (i.e., tide pool, high zone, mid zone, and low zone). Error values indicate 1 SE, and values inside parentheses indicate percentages of quadrats in which respective snail species were found.

Among five snail species, *N*. *funiculata* was the most widespread species across the entire intertidal habitat (found in 66% of quadrats), while *T*. *pellisserpentis* and *N*. *scabricosta* were the least frequently observed species (found in 28% of quadrats). When pooling all quadrats, mean density (no. individual per m^2^ ± 1 SE) for each snail species was as follows: *T*. *pellisserpentis* = 0.7 ± 0.2, *N*. *scabricosta* = 4.1 ± 1.5, *N*. *funiculata* = 23.6 ± 5.1, *P*. *planicostatus* = 19.1 ± 4.6, *C*. *stercusmuscarum* = 21.0 ± 7.1 (Table 1).

### Host selection behaviors in flatworms

There was a significant difference in infestation probability among five snail hosts during host selection trials (F_[__4__,85]_ = 16.5, *P* < 0.0001; Fig 2C). Multiple comparisons of LMM outputs revealed that flatworms preferred snail species in the order of *T*. *pellisserpentis* > *N*. *scabricosta* = *N*. *funiculata* > *P*. *planicostatus* = *C*. *stercusmuscarum* (Fig 2C).

### Host rejection of flatworm infestation

**Fig 3:**
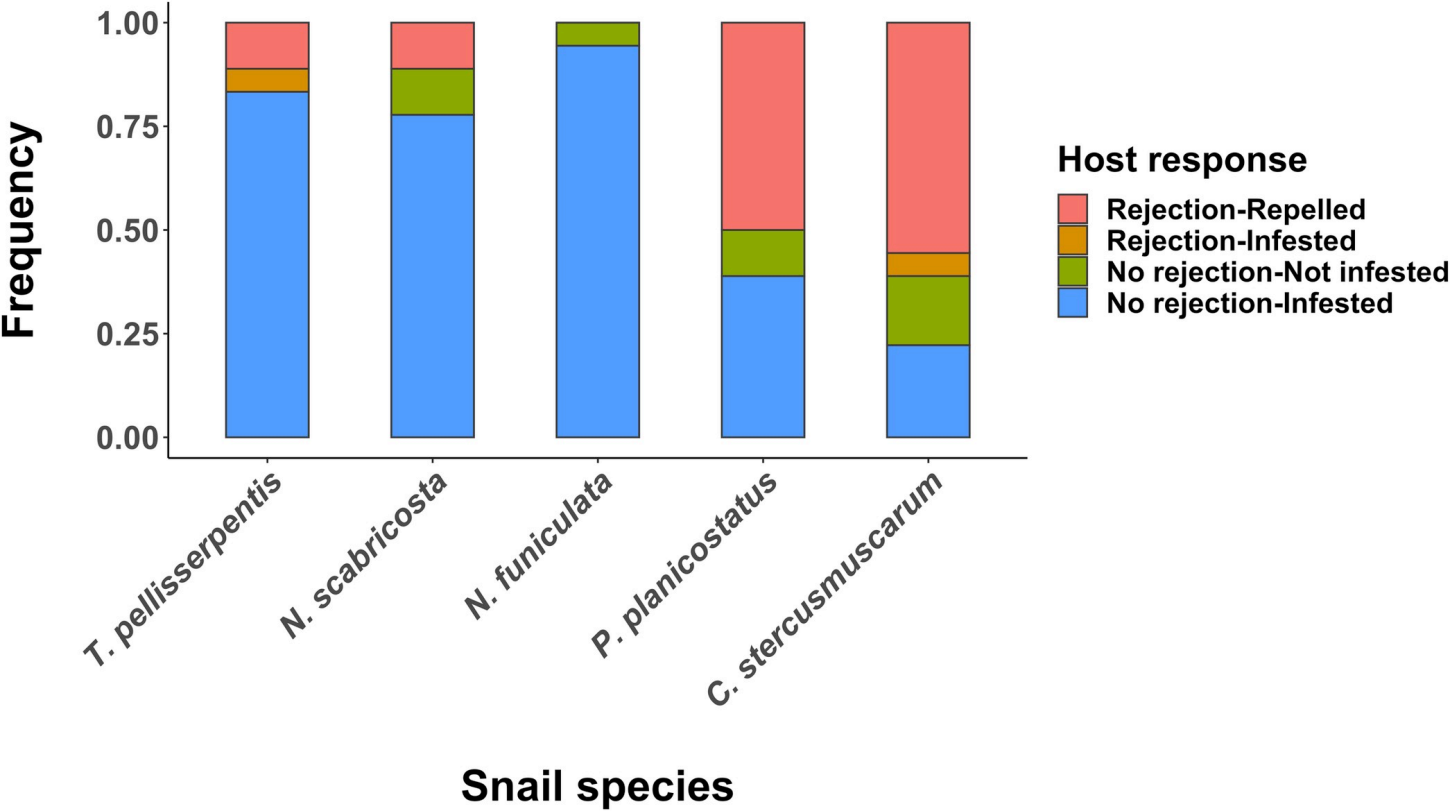
Host rejection behaviors and flatworm infestation success. Frequencies of different snail and flatworm responses during experimental infestation. The responses were classified as: the host rejects and repels the worm (red pink), the host rejects the worm, but the worm successfully infests the host (orange), the host does not reject the worm, but the worm does not infest the host (olive green), and the host does not reject the worm, and the worm successfully infests the host (light blue). Snail and flatworm responses were tracked over the allotted time period of 3 minutes using 18 different individuals for each species.

All snail species except *N*. *funiculata* showed some degree of behavioral rejection when exposed to flatworms (Fig 3). Frequencies in which snail hosts rejected and subsequently repelled flatworms varied among snail species (Logistic regression, χ^2^ = 74.8, df = 4, *P* < 0.0001), being significantly higher in *P*. *planicostatus* (50%) and *C*. *stercusmuscarum* (55.6%) in comparisons to *T*. *pellisserpentis* (11.1%), *N*. *scabricosta* (11.1%), and *N*. *funiculata* (0%) (Holms-corrected *P* < 0.05 for all comparisons) (Fig 3). Conversely, the probability of successful infestation, irrespective of host rejection responses, was significantly higher in *T*. *pellisserpentis* (88.9%), *N*. *scabricosta* (77.8%) and *N*. *funiculata* (94.4%) in comparisons to *P*. *planicostatus* (38.9%) and *C*. *stercusmuscarum* (27.8%) (Logistic regression, χ^2^ = 84.7, df = 4, *P* < 0.0001; Holms-corrected *P* < 0.05 for all comparisons) (Fig 3).

### Host growth and survival following worm infestations

**Fig 4:**
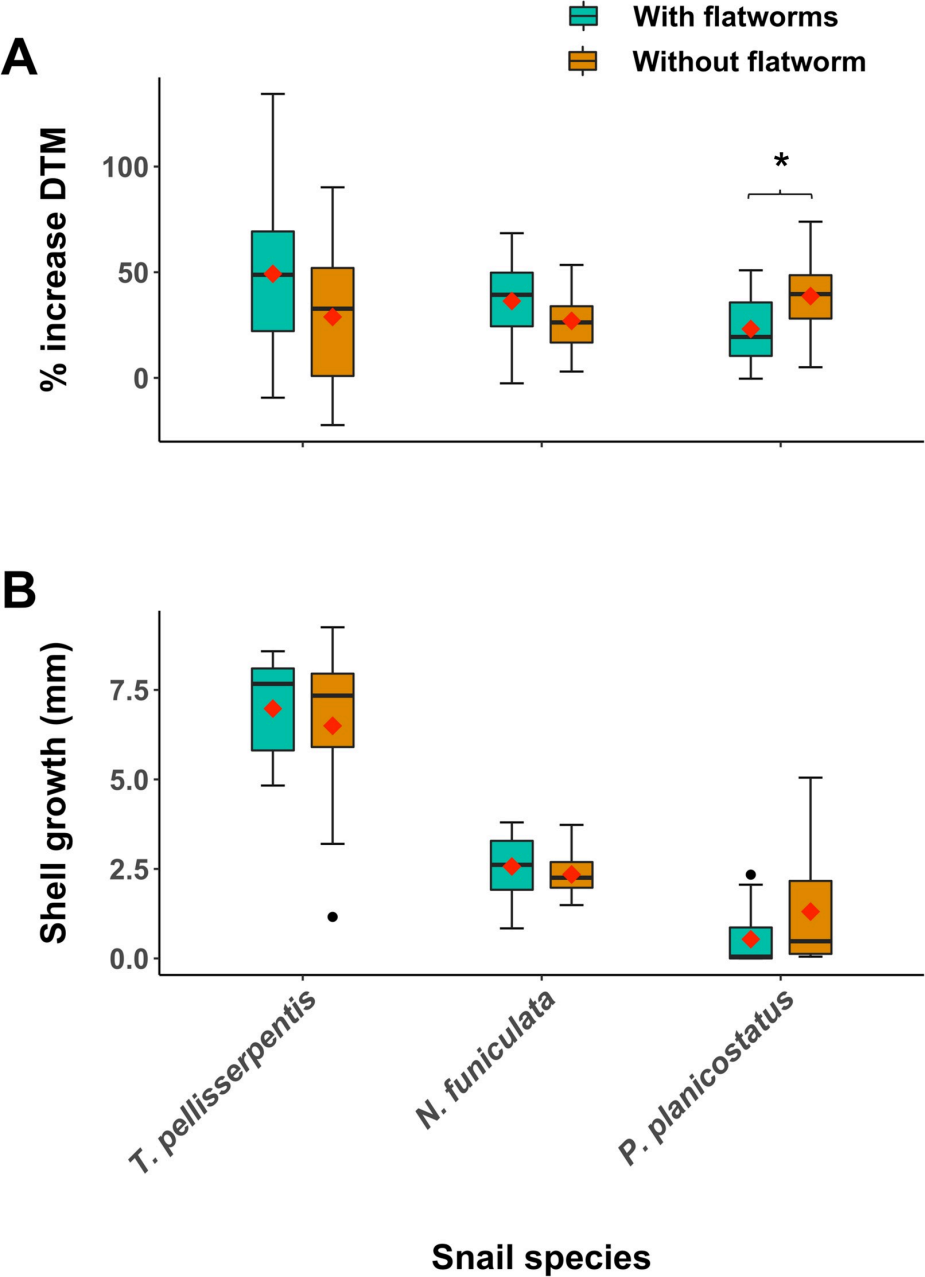
Effect of flatworm infestation on snail growth performances. Box plots of (A) percentage increase in snail dried tissue mass (DTM) and (B) growth in the shell aperture lip (mm) for *Tegula pellisserpentis*, *Nerita funiculata*, and *Planaxis planicostatus* with (turquoise box) and without (dark orange box) flatworms during the field experiment. Mean values for different treatment groups are represented by red diamond symbols. The asterisk (*) indicates a significant difference (P < 0.05).

Flatworms negatively affected the growth of *P*. *planicostatus*, significantly decreasing its DTM (Linear regression GLM; χ^2^ = 8567.2, df = 29, *P* = 0.01, Fig 4A) and marginally decreasing shell growth (χ^2^ = 42.2, df = 29, *P* = 0.08, Fig 4B). Flatworms did not significantly affect the growth of *T*. *pellisserpentis* (Fig 4A and 4B) and marginally increased the DTM of *N*. *funiculata* (χ^2^ = 8753.4, df = 32, *P* = 0.1, Fig 4A). Flatworm infestation did not affect host mortality in all cases. In the treatment group, 23%, 17%, and 0% of the surviving *T*. *pellisserpentis* (n = 13), *N*. *funiculata* (n = 18), and *P*. *planicostatus* (n = 15) had at least one worm residing inside mantle cavity, respectively. No new infestation occurred in the control groups (i.e., snails without flatworm).

### Stable isotope analysis of host and flatworm tissues

**Fig 5:**
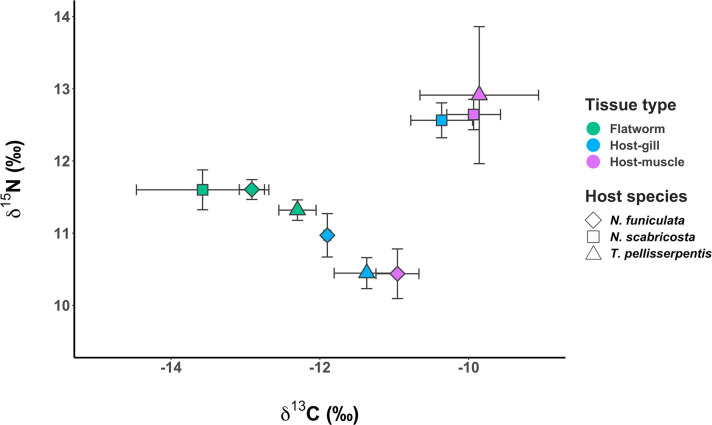
Stable isotope analysis of flatworm and snail tissues. Tissue δ ^13^C and δ ^15^N of flatworms and their snail host species: *Nerita funiculata* (diamond), *N*. *scabricosta* (square), and *Tegula pellisserpentis* (triangle). Different colors represent tissues sampled from flatworm (emerald green), snail gill (light blue), and snail mantle and foot muscle (purple pink). Sample size was n = 4−8 for each snail species × tissue category. Error bars indicate 1 SE.

Flatworm tissues showed limited variation in δ ^15^N (0.0‰−0.3‰) and δ ^13^C (0.6‰−1.28‰) values among different host species categories (Fig 5). Snail gill and muscle tissues had similar δ ^15^N values in *N*. *scabricosta* and *N*. funiculata, but these values varied more in *T*. *pellisserpentis* (~ 2.5‰). Flatworm δ ^15^N values were within − 1.6‰ to 1.2‰ of host gill and muscle δ ^15^N, whereas flatworm δ ^13^C values were generally lower (1.0‰−3.6‰) compared to host δ ^13^C (Fig 5).

## Discussion

This is the first study to examine the host preference of polyclad flatworms in relation to their varying effects on host organisms. Flatworms’ host selection mirrored their field prevalence and infestation density in different snail hosts. However, these patterns were not explained by the relative availability of different hosts as the preferred species (*T*. *pellisserpentis*, *N*. *scabricosta*, and *N*. *funiculata*) were not necessarily more abundant in the field. Therefore, consistent with previous findings by Fujiwara et al. [30], host preference rather than host availability appears to drive the differential infestation patterns by symbiotic flatworms. The mechanism by which flatworms distinguish different hosts is unclear, but it is likely influenced by both chemical [40] and physical cues [30] from snails.

The preferred and less-preferred snail hosts showed marked differences in their behavioral defenses and post-infestation performances. *P*. *planicostatus* and *C*. *stercusmuscarum* frequently rejected and repelled infesting worms, but such responses were reduced or absent in *T*. *pellisserpentis*, *N*. *scabricosta*, and *N*. *funiculata*. Accordingly, flatworms were 39% to 67% less successful when they attempted to infest *P*. *planicostatus* and *C*. *stercusmuscarum* compared to *T*. *pellisserpentis*, *N*. *scabricosta*, and *N*. *funiculata*. As predicted, host species displaying stronger rejection behaviors performed more poorly upon infestation. Flatworms negatively influenced the growth of *P*. *planicostatus* in the field but negligibly affected the growth and survival of *T*. *pellisserpentis* and *N*. *funiculata*, even though we treated the latter two hosts with higher worm loads. These findings are concurrent with predictions that the strength of host counter-defense or resistance may vary depending on the potential costs of hosting a symbiont [41, 42].

We found no evidence to suggest that flatworms are parasitic. Analysis of host and symbiont δ ^15^N revealed that flatworms are unlikely to feed on hosts’ soft tissues. Flatworm δ ^15^N values were well within > 3.4‰ of host gill and muscle δ ^15^N (− 1.6‰ to 1.2‰ differences) [36]. Also, consistent with previous observations [33], we did not find visible tissue damage in the gill and cavity wall of infested snail hosts. These findings suggest flatworms are likely commensal, and their deleterious effects on *P*. *planicostatus* may be a byproduct of the infestation. Because flatworms inhabit the host branchial chamber, they might compete for oxygen with hosts or physically interfere with the hosts’ oxygen uptake, as hypothesized for other gill-dwelling symbionts [43]. *P*. *planicostatus* may have been especially prone to such effects due to its relatively small body size and mantle cavity volume (Table 1) or other anatomical differences with *T*. *pellisserpentis* and *N*. *funiculata* (e.g., gill complex morphology) [44].

Our findings support the hypothesis that flatworm’s host preference could be mediated by the behavioral defense and post-infestation performance of snail hosts. Selecting more defensive *P*. *planicostatus* and *C*. *stercusmuscarum* would decrease the probability of successful infestation when flatworms seek new hosts or leave and re-enter snail hosts during foraging [45]. Flatworms had no direct impact on the survival of *P*. *planicostatus*. However, by negatively affecting its performance, flatworm infestation could render the host more susceptible to predation or physical stress. Host mortality would be detrimental to flatworms in many cases, as they may easily succumb to desiccation and predation outside snail hosts [30]. Conversely, selecting less defensive *T*. *pellisserpentis*, *N*. *scabricosta*, and *N*. *funiculata* would increase the probability of successful worm infestation and re-infestation. These hosts could also provide more stable habitats for flatworms, as they are less likely to be harmed by the infestation.

Differences in host qualities may have further affected the flatworm’s host choice. Among the preferred hosts, flatworms showed the strongest preference for *T*. *pellisserpentis*, which had the largest body size and cavity volume (Table 1). Indeed, it is not uncommon for symbionts to prefer and perform better in larger hosts that offer more generous habitat space and food resources [46, 47]. Flatworms not only attained higher densities but also 2−5 times larger body sizes in *T*. *pellisserpentis* than in other hosts (S1 Table). It is unclear whether flatworms enjoy higher reproductive success in the host species. However, a previous study found a strong correlation between the maternal body size of symbiotic polyclads and their reproductive output [45].

Our results corroborate the growing evidence that interspecific variations in host responses could modulate host preferences in symbioses. In a cleaning mutualism between branchiobdellid worms and freshwater crayfish hosts, the symbionts may prefer host species that are less likely to behaviorally inhibit their colonization (e.g., via grooming activities) [22]. Similarly, in parasitic interactions between parasitoids and insect hosts [26] or freshwater trematodes and amphibians [19], the symbionts may prefer host species with less effective immune defenses. Moreover, some parasites and pathogens may avoid host species in which the infection kills the host too quickly and impede cross-host transmission [20]. Symbionts commonly interact with hosts that are phylogenetically distinct or possess different morphological, behavioral, and physiological traits [48–50], which may contribute to varying host response patterns. Therefore, we predict that host response variabilities would play broad and significant roles in the evolution and maintenance of host preference.

Previous studies considered symbiotic polyclads as commensal [28, 45] or parasites that prey on host tissues or embryos [51, 52]. However, their effects on host organisms have rarely been tested using empirical approaches. The results from our field experiment and stable isotope analysis agree with Fujiwara et al. [45] that flatworms live as commensal of intertidal snails. Flatworms may use snails as mobile homes and feed on similar food sources as the hosts (e.g., algae and other periphytic materials) [53], while it is unclear whether they obtain food mainly inside or outside their hosts. Future work should more broadly examine the symbiotic nature and evolutionary history between these organisms. Indeed, similar species associations have been reported across different continents (e.g., America, Asia, and Africa) and from various families of gastropods and polyclads [28, 33].

Our study is not without caveats. In the host selection experiment, we exclusively used flatworms collected from three commonly infested host species, which could have biased our findings. Symbionts can imprint to host chemical or visual cues upon colonization, which may influence their future host choice [3, 54]. Hence, a proper test of host preference should either control the source hosts (e.g., introducing the same number of worms collected from each host species) or use “virgin” symbionts with no prior interactions with hosts. Neither was feasible in our study due to difficulties in obtaining unsettled flatworm larvae and collecting sufficient adult worms from all snail species. However, we note that flatworms were 90% to 130% more likely to choose *T*. *pellisserpentis* than *N*. *scabricosta* and *N*. *funiculata*, although our study used an equal number of worms collected from these hosts. Thus, our results maintain that a pre-existing host preference would govern the flatworm’s host choice.

In conclusion, we demonstrate that host response traits could modulate host choices in symbiotic interactions. As predicted, flatworms’ host preference was positively associated with the reduced host behavioral defense and the hosts’ ability to support flatworm populations without being harmed. We encourage future studies to more explicitly consider host response variability when examining multi-species symbioses. These tests will improve our theoretical and empirical understanding of complex biological processes shaping host preference.

## Supporting information

S1 Fig Experimental cages used in the field experiment. The cages (containing experimental snails) deployed at the rocky intertidal habitats within Smithsonian Tropical Research Institute’s Punta Culebra Nature Center (PCNC), Panama.
(TIF)

S1 Table Flatworm biomass in different snail hosts. Mean blotted wet mass (BWM; mg) of total and individual flatworms collected from different snail species (2012 field surveys). Error values indicate 1 SE and values inside parentheses represent sample sizes (i.e., number of snails dissected).
(DOCX)

S1 Data Supplementary data (flatworm infestation data, host choice and behavioral response data, host growth and survival data, and stable isotope data). (XLSX)
