# Supplementary figures and images for: Variable host responses mediate host preference in marine flatworm−snail symbioses
Source: PLoS One. 2021 Mar 2;16(3):e0247551. doi: 10.1371/journal.pone.0247551 (PMC7924752; doi:10.1371/journal.pone.0247551)

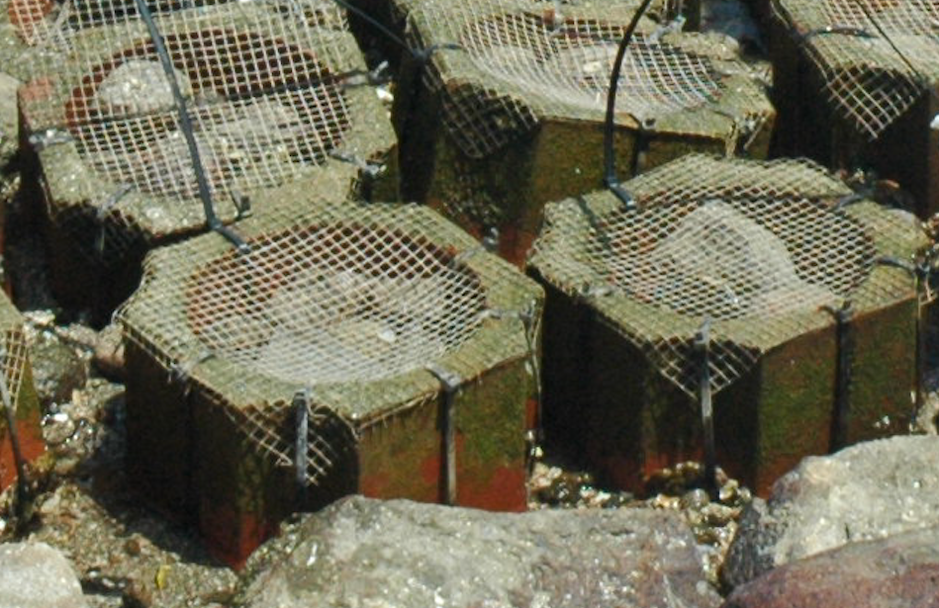

Supplement: S1 Fig — The cages (containing experimental snails) deployed at the rocky intertidal habitats within Smithsonian Tropical Research Institute’s Punta Culebra Nature Center (PCNC), Panama. (TIF) [file pone.0247551.s001.tif]
