# Supplementary material for: Variable host responses mediate host preference in marine flatworm−snail symbioses
Source: PLoS One. 2021 Mar 2;16(3):e0247551. doi: 10.1371/journal.pone.0247551 (PMC7924752; doi:10.1371/journal.pone.0247551)
Supplement: S1 Table — Mean blotted wet mass (BWM; mg) of total and individual flatworms collected from different snail species (2012 field surveys). Error values indicate 1 SE and values inside parentheses represent sample sizes (i.e., number of snails dissected). (DOCX) [file pone.0247551.s002.docx]

| Snail species | Mean blotted wet mass (mg) | |
| --- | --- | --- |
|  | Total worm mass | Individual worm mass |
|  |  |  |
| *T. pellisserpentis* | 18.8 ± 2.0 mg (80) | 4.3 ± 2.0 mg (80) |
| *N. scabricosta* | 9.7 ± 2.4 mg (46) | 2.2 ± 0.2 mg (46) |
| *N. funiculata* | 3.0 ± 0.6 mg (28) | 1.2 ± 0.2 mg (28) |
| *P. planicostatus* | 0.8 ± 0.3 mg (3) | 0.8 ± 0.3 mg (3) |
| *C. stercusmuscarum* | 0.9 ± 0.0 mg (1) | 0.9 ± 0.0 mg (1) |

**S1 Table.**
